# COVID-19 focuses attention on medical-dental collaboration

**DOI:** 10.5116/ijme.60e2.f802

**Published:** 2021-07-29

**Authors:** Louay Jaber, Steph Smith

**Affiliations:** 1 Department of Biomedical Sciences, College of Dentistry, Imam Abdulrahman Bin Faisal University, Saudi Arabia; 2 Department of Preventive Dental Sciences, College of Dentistry, Imam Abdulrahman Bin Faisal University, Saudi Arabia

**Keywords:** COVID-19, medical-dental collaboration, dental education, Saudi Arabia

## Introduction

Dentistry is perceived as the clinical practice of restoring teeth. However, a report released in 1995 from the Institute of Medicine (IOM), “Dental Education at the Crossroads: Challenges and Change”, states the need to integrate dentistry with medical healthcare delivery systems at all levels.^1^ In spite of this recommendation, the schism between medical and dental professionals continues to segregate the two professions,^2^ with the prevailing Coronavirus Disease-2019 (COVID-19) pandemic further highlights the shortcomings of medical-dental collaboration. The recent pandemic has presented an immense challenge to public health officials who have witnessed an escalating demand for medical support in a variety of healthcare settings. Therefore, healthcare professionals, such as retired physicians, nurses and other ancillary staff, have been approached to help combat this devastating disease. However, to the best of the authors’ knowledge, no official call was made to enlist dentists to provide assistance during these difficult times. This intentional exclusion of dentists could negatively impact diagnosis, triage, and the clinical management of responsibilities recommended by the World Health Organization (WHO).^3^ In addition, this raises fundamental questions and concerns regarding the reason(s) for excluding dental professionals from participating in these collaborative efforts.

In the light of the above, the present article provides some perspectives on the possible role and duties of dentists for tackling the COVID-19 crisis and the crucial need to implement the recommendations of the IOM regarding collaborative activities of medical and dental clinicians.

### Potential Role of Dentists during Coronavirus-2019 Pandemic

The American Dental Education Association has long advocated that dental offices should serve as a “portal to primary care”, especially for those patients that do not attend on a regular basis.^4^  Hence, it seems plausible that dentists could serve as a gateway for direct or indirect care during the present pandemic, for example, screening and/or testing patients for the COVID-19, participating in activities recommended by senior physicians, including telephone triage and/or assessment of symptomatic patients, administrative tasks, online and outreach for residents of nursing homes, supportive advice for residential communities to cope with isolation and loneliness, or assisting local practices by producing patient education materials and information sheets using local and/or regional resources.^5^ This envisaged participation of dental healthcare professionals could potentially decrease the workload of medical staff, thereby allowing them more time to deal with frontline emergencies.

Unfortunately, the separation between medical and dental professionals continues despite the earlier recommendations of the IOM regarding the crucial need for coordinated patient care and communication among healthcare professionals.^6^^,^^7^ This would suggest the necessity to re-encourage an effective partnership between medical and dental stakeholders to overcome differences and fully implement the IOM recommendations.

### Challenges Facing Medical-Dental Collaboration

The 2003 Institute of Medicine report “Health Professions Education: A Bridge to Quality”, focuses on integrating a core set of competencies for healthcare professional education, including patient-centered care, interdisciplinary teams, evidence-based practice, quality improvement, and informatics. Furthermore, it envisages collaboration as a fundamental and critically imperative prerequisite for the effective functioning of the healthcare community and also to act as a benchmark on which all systems should be grounded.^8^ Since the publication of this report, substantial progress has been made for addressing the IOM core recommendations by administrative officials in both medical and dental schools who have shown considerable interest in interprofessional education activities. Nevertheless, hurdles do exist, for instance, the lack of time in the curriculum, overcoming interpersonal conflicts, faculty training facilities and budget limitations.^2^^,^^9^ Also, the separate training of physicians and dentists severely compromises their ability to work as a collaborative team. Hence, integrating educational systems between different health professional schools is essential for overcoming these weaknesses and strengthening collaborative endeavours. For example, the knowledge and training of physicians (general medical practitioners) for promoting oral health care for patients with chronic diseases, such as diabetes, is limited.^10^ On the other hand, insufficient medical training at the dental undergraduate level predisposes general dentists to be incompetent in hospital settings, which could be a major reason for excluding them during the present COVID-19 pandemic.

In this context, it is interesting to lay emphasis upon the successful past experience of medical-dental integration whereby dentistry is considered as a specialty of medicine.^11^^,^^12^ The graduate of the latter (stomatology) has the advantage of being versed with a medical background and confident in a medical environment, whereas the graduate of a purely dental program (odontology) has knowledge that is mainly limited to the oral cavity, and hence, may feel inept or outside their comfort zone when confronted with medical challenges. Therefore, a more comprehensive and diverse educational system is proposed that combines medical and dental training that would be pivotal for enhancing collaboration.

In summary, the present and incessant COVID-19 pandemic, which continues to ravage the world, has highlighted the shortcomings of medical-dental collaboration, which might negatively impact patient care. Therefore, it is strongly recommended that dental professionals should be mobilized to assist in direct or indirect patient care so as to free up their medical colleagues to deal with other frontline matters. Also, it is necessary that medical and dental education experts, together with public health officials, continue to incorporate medical and dental educational systems at all levels, with the aim of ensuring that future dentists and physicians collaborate to provide enhanced patient care, whether in private practices or other healthcare settings. This is not a preference but an ethical obligation to promote medical-dental collaboration that ultimately benefits both professions, and most importantly, the patient.

### Conflicts of Interest

The authors declare that they have no conflict of interest.

### Acknowledgment

The authors would like to thank Dr Irfan Ahmad for editing the manuscript.
